# Elevated CO_2_ Levels Delay Skeletal Muscle Repair by Increasing Fatty Acid Oxidation

**DOI:** 10.3389/fphys.2020.630910

**Published:** 2021-01-21

**Authors:** Ermelinda Ceco, Diego Celli, Samuel Weinberg, Masahiko Shigemura, Lynn C. Welch, Lena Volpe, Navdeep S. Chandel, Ankit Bharat, Emilia Lecuona, Jacob I. Sznajder

**Affiliations:** ^1^Division of Pulmonary and Critical Care Medicine, Feinberg School of Medicine, Northwestern University, Chicago, IL, United States; ^2^Division of Thoracic Surgery, Feinberg School of Medicine, Northwestern University, Chicago, IL, United States

**Keywords:** hypercapnia, chronic obstructive pulmonary diseases, muscle differentiation, β-Oxidation, cardiotoxin

## Abstract

Muscle dysfunction often occurs in patients with chronic obstructive pulmonary diseases (COPD) and affects ventilatory and non-ventilatory skeletal muscles. We have previously reported that hypercapnia (elevated CO_2_ levels) causes muscle atrophy through the activation of the AMPKα2-FoxO3a-MuRF1 pathway. In the present study, we investigated the effect of normoxic hypercapnia on skeletal muscle regeneration. We found that mouse C2C12 myoblasts exposed to elevated CO_2_ levels had decreased fusion index compared to myoblasts exposed to normal CO_2_. Metabolic analyses of C2C12 myoblasts exposed to high CO_2_ showed increased oxidative phosphorylation due to increased fatty acid oxidation. We utilized the cardiotoxin-induced muscle injury model in mice exposed to normoxia and 10% CO_2_ for 21 days and observed that muscle regeneration was delayed. High CO_2_-delayed differentiation in both mouse C2C12 myoblasts and skeletal muscle after injury and was restored to control levels when cells or mice were treated with a carnitine palmitoyltransfearse-1 (CPT1) inhibitor. Taken together, our data suggest that hypercapnia leads to changes in the metabolic activity of skeletal muscle cells, which results in impaired muscle regeneration and recovery after injury.

## Introduction

Hypercapnia, elevated CO_2_ levels in tissues and blood, is seen in patients with inadequate alveolar gas exchange, such as chronic obstructive pulmonary disease (COPD), and it is associated with worse clinical outcomes (Yang et al., 2015; Fazekas et al., 2018). Recently, several reports suggested that hypercapnia, independently of pH and oxygen levels, activates distinct signaling pathways, with deleterious effects on the lung epithelia, mature muscle fibers, adipocytes, and the innate immune system (Vadasz et al., 2008; Cummins et al., 2010; Gates et al., 2013; Jaitovich et al., 2015; Kikuchi et al., 2017; Shigemura et al., 2017, 2018; Korponay et al., 2020).

Patients with COPD develop muscle atrophy, and their skeletal muscle function continues to decline over time, affecting their quality of life (Debigare et al., 2001; Wust and Degens, 2007; Gea et al., 2016; Barreiro and Jaitovich, 2018). Even though COPD skeletal muscle dysfunction may affect both ventilatory and limb muscles, the latter are usually more severely affected (Barreiro and Jaitovich, 2018). A multicenter European-based study showed that quadriceps muscle dysfunction occurred in approximately one-third of the COPD patients, even at the early stages of their disease (Seymour et al., 2010; Barreiro and Jaitovich, 2018). The prevalence of muscle weakness among the patients did not significantly correlate with disease severity but was associated with body mass index, airflow obstruction, dyspnea, exercise capacity, and dyspnea scores (Seymour et al., 2010; Barreiro and Jaitovich, 2018).

Human skeletal muscle is about 40% of the body mass and is formed by a bundle of contractile multinucleated muscle fibers, resulting from the fusion of myoblasts (Laumonier and Menetrey, 2016). Healthy skeletal muscle undergoes continuous and repeating cycles of damage and repair to maintain muscle mass (Tidball, 2011; Laumonier and Menetrey, 2016). As such, skeletal muscle repair is essential for recovery from physical or chemical insult (Laumonier and Menetrey, 2016). Skeletal muscles rely on satellite cells for repair, which are stem cells that lie dormant beneath the basal lamina. Once satellite cells become activated, they proliferate, differentiate, and fuse with mature muscle fibers to overcome the damaged state (Snijders et al., 2015; Laumonier and Menetrey, 2016).

Changes in metabolism may regulate signaling pathways and gene expression dictating biological outcomes (Chandel, 2015; van der Knaap and Verrijzer, 2016; Schvartzman et al., 2018; Maniyadath et al., 2020). Due to its role in support and body movement, skeletal muscle function requires high rates of cellular metabolism and energy production (Rolfe and Brown, 1997; Ceco et al., 2017). Cellular ATP is derived from glycolysis, fatty acid oxidation, and proteolysis, and skeletal muscle relies on each of these energy-generating processes (Koopman et al., 2014; Ceco et al., 2017). However, increased use of fatty acids as a source of carbons to feed the TCA cycle by skeletal muscle eventually results in atrophy, as seen in patients with cancer and cachexia (Fukawa et al., 2016). Metabolic status has also been shown to impact satellite cell myogenic activity (Ryall et al., 2015; Ceco et al., 2017).

We have reported that high CO_2_ induces skeletal muscle atrophy through the activation of the E3 ubiquitin ligase muscle RING-finger protein-1 (MuRF-1; Jaitovich et al., 2015). In the present study, we investigated the effect of elevated CO_2_ on skeletal muscle repair and found that hypercapnia induces a metabolic maladaptation, resulting in increased fatty acids β-oxidation and mitochondrial respiration leading to impaired myoblast differentiation.

## Materials and Methods

### C2C12 Myoblast Culture, Differentiation, and CO_2_ Exposure

**Figure 1:**
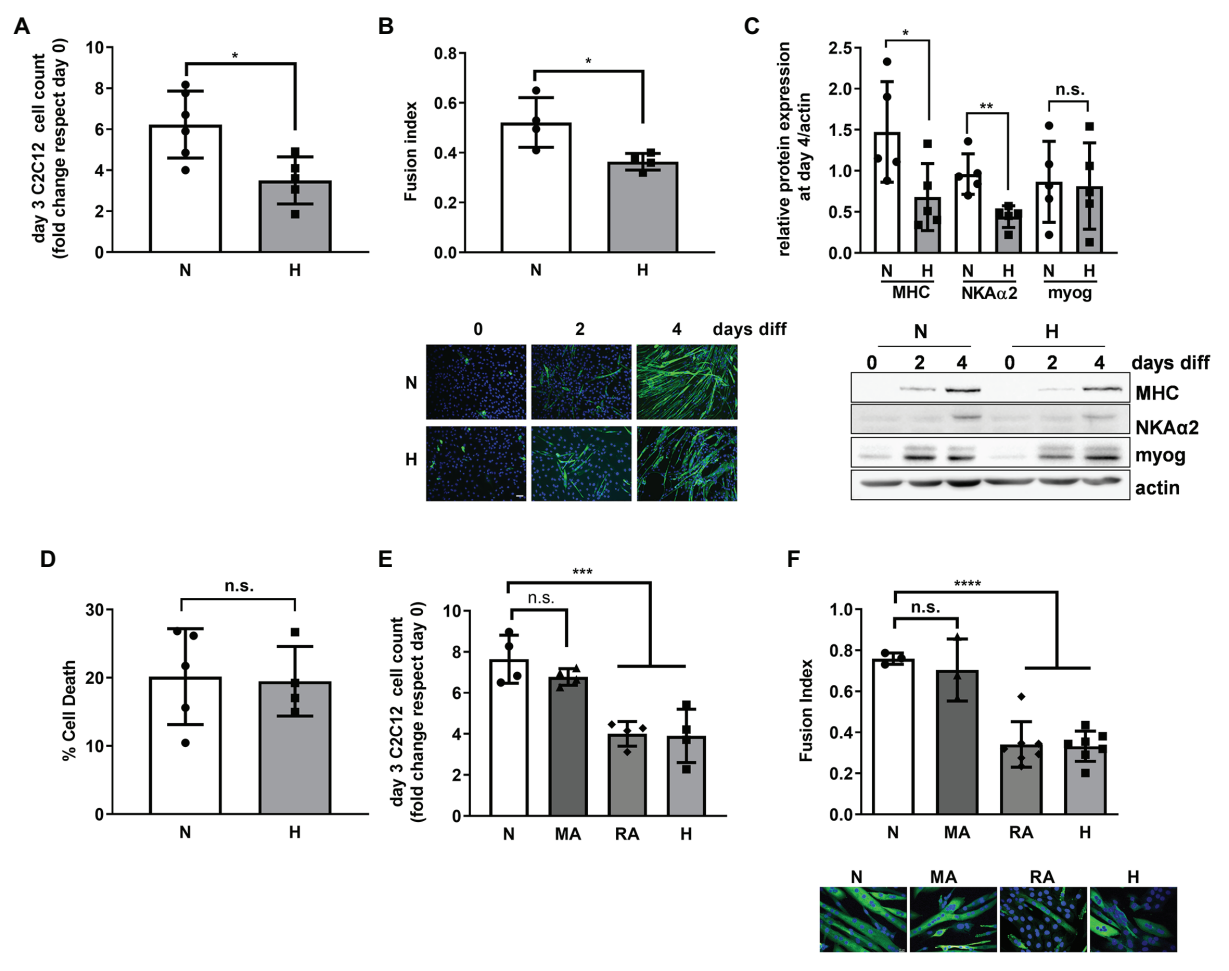
High CO_2_ impairs the differentiation of C2C12 myoblasts into myotubes, independently of pH. **(A)** Same amounts of C2C12 myoblasts were seeded in 3.5 cm plates and grown in normal (N) or high CO_2_ conditions (H) for 3 days. Graph shows fold change of cell count number (*n* = 5). **(B)** C2C12 myoblasts were exposed to normal (N) or high CO_2_ conditions (H) for 3 days and allowed to differentiate into myotubes in normal CO_2_ conditions up to 4 days. Upper panel: graph showing fusion index (*n* = 4). Lower panel: representative immunofluoresecent images from C2C12 myoblasts at 0, 2, and 4 days of differentiation showing myosin heavy chain (MHC; green) expression and nuclei (blue). Scale bar: 100 μm. **(C)** C2C12 myoblasts were exposed to normal (N) or high CO_2_ conditions (H) for 3 days and allowed to differentiate into myotubes in normal CO_2_ conditions for 4 days. Upper panel: graph showing relative values of MHC, Na,K-ATPase α2-subunit (NKAα2), and myogenenin (myog; *n* = 5). Lower panel: representative Western blots. **(D)** Same amount of C2C12 myoblasts were seeded in 3.5 cm plates and grown in normal (N) or high CO_2_ conditions (H) for 3 days. Graph shows % of cell death (*n* = 4–5). **(E)** Same amount of C2C12 myoblasts were seeded in 3.5 cm plates and grown in normal CO_2_ (N), metabolic acidosis (MA), respiratory acidosis (RA) or high CO_2_ conditions (H) for 3 days. Graph shows fold change cell count number (*n* = 4). **(F)** C2C12 myoblasts were exposed to normal CO_2_ (N), MA, RA, or high CO_2_ conditions (H) for 3 days and allowed to differentiate in normal CO_2_ conditions up to 4 days. Upper panel: graph showing fusion index (*n* = 3–6). Lower panel: representative immunofluoresecent images from C2C12 myoblasts at the different conditions showing MHC (green) expression and nuclei (blue). Scale bar: 20 μm. Data from **(A–D)** were analyzed by unpaired *t*-test. Data from **(E,F)** were analyzed by one-way ANOVA followed by Tukey’s multiple comparison test. ^*^p < 0.05; ^**^p < 0.01; ^***^p < 0.001; ^****^p < 0.001; n.s: not significant.

C2C12 mouse myoblasts were obtained from ATCC (#CRL1772) and cultured under normoxia normocapnia (control CO_2_; pCO_2_: 30–40 mmHg, pH: 7.4), normoxia hypercapnia (high CO_2_; pCO_2_: 100–120 mmHg, pH: 7.35–7.44), normoxia metabolic acidosis (pCO_2_: 30–40 mmHg, pH: 7.15–7.24), or normoxic respiratory acidosis (pCO_2_: 100–120 mmHg, pH: 7.15–7.24). The value of pCO_2_: 100–120 mmHg for hypercapnia conditions are based in our previous publications (Vohwinkel et al., 2011; Shigemura et al., 2019). The buffering capacity of the culture medium was modified by changing its initial pH with Tris-MOPS solution to obtain a pH of 7.4 at the various CO_2_ levels as before described (Vohwinkel et al., 2011; Shigemura et al., 2018). The desired CO_2_ and pH levels were achieved by equilibrating the medium overnight in a humidified chamber (C-Chamber, BioSpherix Ltd.). The atmosphere of the C-Chamber was controlled with a ProCO_2_ carbon dioxide controller (BioSpherix Ltd.). In this chamber, cells were exposed to the desired pCO_2_ while maintaining 21% O_2_ balanced with N_2_. Before and after CO_2_ exposure, pH, pCO_2_, and pO_2 Le_vels in the medium were measured using a Stat Profile pHOx blood gas analyzer (Nova Biomedical Corp.). Experiments were started by replacing the culture medium with the CO_2_-equilibrated medium and incubating in the C-Chamber for the desired time. The myoblasts were seeded at desired concentrations, taking in account our finding that proliferation is decreased in high CO_2_ conditions (Figure 1A) and exposed to either normal or high CO_2_ for the required length. For myotubes experiments, after initial exposure, media were changed to low serum (2% horse serum, Corning, #35030 V) and differentiated in normal CO_2_ conditions for 2 or 4 days. For the rescue experiments, 10 μM of etomoxir (Tocris, #4539) was added to either the normocapnia or hypercapnia growth media during the 3 days of pre-exposure and then switched to low-serum containing media for 2 or 4 days to induce myoblast differentiation.

### Immunocytochemistry

C2C12 myoblasts were grown on glass cover slips and pre-exposed to either normal or high CO_2_ for 3 days. Myoblasts were differentiated in low-serum containing media in normocapnia control conditions. Cells were fixed using 4% formaldehyde and incubated with primary antibody anti-pan myosin heavy chain (Developmental Studies Hybridoma Bank (DSHB) #MF20), and secondary antibody Alexa Fluor-488 goat anti-mouse (Thermo Fisher, #A11001) and images were acquired using Zeiss Axiocam epifluorescence microscope with a 20X objective. Hoechst 33342 was used to stain nuclei (Themo Fisher, #H3570).

### Fusion Index

Fusion index was quantified as the number of nuclei inside the myotube normalized by the total number of nuclei as described previously (Nishiyama et al., 2004).

### Western Blot Analysis

C2C12 myoblasts or myotubes were homogenized in Lysis Buffer (Cell Signaling Technology, #9803 s). Protein concentrations were determined by Bradford assay. Proteins were separated by sodium dodecyl sulfate polyacrylamide gel electrophoresis (SDS-PAGE), transferred to nitrocellulose membranes, immunoblotted, and visualized by chemiluminescence following the manufacturer’s instructions as previously described (Jaitovich et al., 2015). The following commercially available antibodies were used for Western blotting: myosin heavy chain (MHC; DSHB, #MF20), N,K-ATPase α2 (Millipore Sigma, #07-674), mouse monoclonal myogenin (F5D; Santa Cruz Biotechnology, #sc-12,732), rabbit phosphorylated-AMP-activated kinase (pAMPK; Thr-172; Cell Signaling, #2535 s), rabbit AMPK (Cell Signaling, #2532 s), rabbit phosphorylated acetyl-CoA carboxylase (pACC; Ser-79; Cell Signaling, #3661 s), and rabbit actin (Sigma, #A2066). Primary antibodies were detected with horseradish peroxidase-conjugated secondary antibodies (Cell Signaling, #7074 s and #7076 s). Quantification of protein levels was performed by densitometric scanning with ImageJ 1.29X (National Institutes of Health).

### Cell Death

Cell viability was measured by acridine orange (AO)/propidium iodine (PI) viability dye (Nexcelom Biosciences, #C52-0106) using the Cellometer K2 (Nexcelom Biosciences).

### Metabolomic Analysis

C2C12 myoblasts were exposed to normal or high CO_2_ for 3 days, trypsinized, and 3–5 × 10^6^ cells per condition (in biological replicates) were used for the metabolic analysis by Metabolon (Durham, NC). The samples were treated with methanol under vigorous shaking for 2 min to remove protein-dissociated small molecules bound to protein or trapped in the precipitated protein matrix and to recover chemically diverse metabolites and then centrifuged. The resulting extracts, including controls, were then analyzed by UPLC-MS/MS. Raw data are given in Supplementary Table 1. Prism (GraphPad; Version 8.4.3) and MetaCore (Thomson Reuters) were used for analysis.

### Mitochondrial Respiration Analysis

The SeaHorse technology (Agilent Technologies; model XFe96) was used to measure the rate of change of dissolved O_2_ in media surrounding the C2C12 myoblasts exposed to normal or high CO_2_ for 3 days in the presence or absence of 10 μM of etomoxir. About 35,000 cells per well were plated for each condition, in a 96-well format, using Cell-Tak (Corning, # 40240) as instructed by the manufacturer to achieve rapid attachment to the plate. For the XFe96 assay, cells were equilibrated with DMEM lacking bicarbonate, with low phenol red and supplemented with 4 mM glutamine, 1 mM pyruvate, and 25 mM glucose at 37°C for 1 h in a non-CO_2_ incubator. A concentration of 2 μM oligomycin was used to block ATP synthase: 0.5 μM of FCCP was used as an uncoupler to measure maximal oxygen consumption rate, and a concentration of 0.5 μM of antimycin and rotenone was used to suppress the oxygen consumption from the mitochondria. Measurements are reported in pmol/min for oxygen consumption.

### Animals

Adult (9–11 weeks old) male C57Bl/6 mice were obtained from The Jackson Laboratory (Bar Harbor, ME). Only male mice were used as the differences in weight made the cross-sectional area (CSA) comparisons not possible. All animals were provided with food and water *ad libitum*, maintained on a 14-h light/10-h dark cycle, and handled according to National Institutes of Health guidelines. All of the procedures involving animals were approved by the Northwestern University Institutional Animal Care and Use Committee (#IS00000245). For high CO_2_ exposure, animals were maintained in a C-Shuttle Glove Box (BioSpherix Ltd., Lacona, NY) for 21 days. The chamber’s atmosphere was continuously monitored and adjusted with ProOx/ProCO_2_ controllers (BioSpherix Ltd.) in order to maintain 10% CO_2_ and 21% O_2_, with a temperature of 20–26°C and a relative humidity between 40 and 50%. These settings resulted in an arterial partial pressure of carbon dioxide (paCO_2_) of ∼75 mmHg and arterial partial pressure of oxygen (paO_2_) of ∼100 mmHg, whereas in animals maintained in room air paCO_2_ was ∼40 mm Hg and paO_2_ was ∼100 mmHg (Jaitovich et al., 2015). None of the mice developed appreciable distress. After 21 days of exposure, the animals were sacrificed with Euthasol (pentobarbital sodium/phenytoin sodium), and the skeletal muscles were excised and fixed in 10% formalin for histological analyses.

### Skeletal Muscle Injury

Ten microliter of 10 μM cardiotoxin (Millipore Sigma, #217503) in sterile phosphate buffered saline (PBS) was injected into the left tibialis anterior (TA) muscle at day 0. The right TA muscle was mock injected with PBS alone. The mice were then placed into the hypercapnia chamber and exposed to 10% CO_2_ for 21 days. At day 21, the mice were sacrificed and muscles were quickly harvested for analysis.

For the rescue experiments, the same procedure was applied for the skeletal muscle injury, but at day −3, and at day 0, 90 μl of 20 μM etomoxir (25 mg/kg) in PBS or PBS alone was injected intraperitoneally every other day until the mice were sacrificed at day 21.

### Histological Analysis

For histological analysis, 8-μm-thick paraffin-embedded sections of TA muscle were stained with H&E at the Northwestern University Mouse Histology and Phenotyping Laboratory. Images were acquired using Zeiss Axiocam epifluorescence microscope with a 10X objective and recorded and processed using AxioVision 2.0. Images were analyzed using ImageJ, version 1.50i, and statistical analyses were performed using Prism (GraphPad; Version 8.4.3). All analyses were performed blindly. Fiber size was studied by measuring the fibers’ minimal inner diameter (at least 100 fibers per muscle), defined as the minimum diameter from inner border to inner border, passing through the center of the muscle fiber as previously described by Jaitovich et al. (2015). CSA was calculated using this diameter, and results were expressed as mean CSA ± SD and as percentage of fibers distributed by size. Centralized nuclei were counted and related to the number of fibers counted on the same field as before described by Jaitovich et al. (2015).

### Statistics

Data are expressed as the mean ± SD. When comparisons were performed between two groups, significance was evaluated by unpaired two-tailed Student’s *t*-test. When more than two groups were compared, one-way ANOVA was performed, followed by Tukey’s post-test, using GraphPad Prism software. Results were considered significant when *p* < 0.05.

## Results

### High CO_2_ Impairs the Differentiation of C2C12 Myoblasts, Independently of pH

To repair a skeletal muscle lesion, satellite cells must be activated, proliferate, and differentiate (Snijders et al., 2015; Laumonier and Menetrey, 2016). We used the C2C12 murine cell line for our *in vitro* studies, a well-established model for the study of muscle regeneration and differentiation (Yaffe and Saxel, 1977; Lawson and Purslow, 2000; Murphy et al., 2016). Exposure of C2C12 myoblasts to high CO_2_ showed decreased proliferation as compared to myoblasts exposed to normal CO_2_, which is in agreement with our previous report, showing decreased proliferation of both epithelial cells and fibroblasts (Vohwinkel et al., 2011; Figure 1A). To determine whether high CO_2_ also affects C2C12 differentiation, we pre-exposed C2C12 myoblasts for 3 days to high CO_2_ or normal CO_2_ and induced them with low serum to differentiate in control conditions. We found that C2C12 myoblasts pre-exposed to high CO_2_ have impaired differentiation, determined by a decreased fusion index (Figure 1B), as well as a decreased expression of differentiation markers like MHC and Na,K-ATPase α2-subunit (Ladka and Ng, 2000; Tsukamoto et al., 2018) with no changes in myogenin expression (Figure 1C). The impairment in C2C12 myoblasts differentiation was not due to increased cell death (Figure 1D).

We sought to determine whether the impaired proliferation and differentiation observed in high CO_2_ conditions was pH dependent. We exposed C2C12 myoblast to metabolic acidosis and respiratory acidosis and found that both impaired C2C12 proliferation and differentiation were independent of pH (Figures 1E,F). Taken together, these data suggest that increased levels of CO_2_ impairs C2C12 myoblast proliferation and differentiation independently of cell death and changes in pH.

### High CO_2_ Induces Metabolic Changes and Increases Mitochondrial Respiration in C2C12 Myoblasts

**Figure 2:**
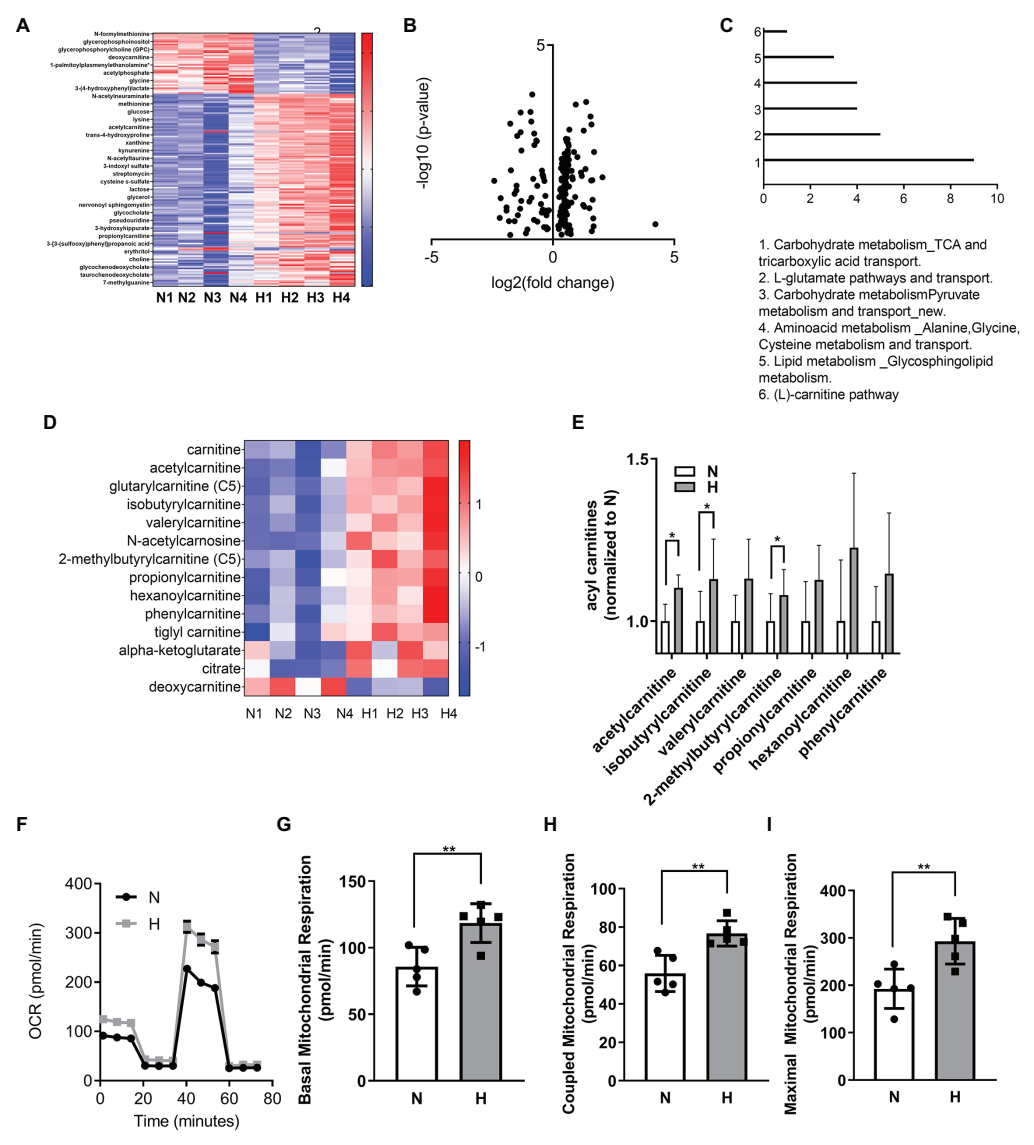
High CO_2_ induces metabolic changes and increases mitochondrial respiration in C2C12 myoblasts. **(A–E)** Metabolomic profiling of C2C12 myoblasts exposed for 3 days to normal (N) or high CO_2_ (H) conditions. **(A)** Heatmap of top most significantly differentially expressed metabolites ranked by FDR *q*-value. Both samples and metabolites are grouped by hierarchical clustering. **(B)** Volcano plot representation of differential metabolites determined under the conditions of fold change ≥2 and false discovery rate-adjusted the value of *p* threshold≤0.05. The value of *p* are transformed by −log10 so that the more significantly different metabolites (with smaller value of *p*) are higher on the y-axis. The fold change is log-transformed so that negative values represent a decrease in metabolite levels and positive values represent an increase in metabolite levels. **(C)** Functional enrichment analysis of metabolic processes was performed with MetaCore. Graph represents the top 6 metabolic processes. **(D)** Heatmap of selected components of carnitine and TCA pathway metabolites. **(E)** Normalized counts of acyl carnitines in normal and high CO_2_ conditions. **(F–I)** Mitochondrial respiration was measured using Seahorse technology to detect the oxygen comsuption rate (OCR) values in C2C12 myoblasts exposed for 3 days to normal (N) or high CO_2_ (H) conditions. **(F)** Graph shows a representative OCR after treatment with oligomycin, FCCP, and rotenone. **(G)** Graph shows basal mitochondrial respiration. **(H)** Graph shows coupled mitochondrial respirationb. **(I)** Graph shows maximal mitochondrial respiration. Data from **(E–I)** were analyzed by unpaired *t*-test. ^*^p < 0.05; ^**^p < 0.01.

We performed metabolic analysis in C2C12 myoblasts exposed to elevated CO_2_ for 3 days to determine whether changes in metabolic processes in the myoblasts exposed to high CO_2_ might explain the impaired differentiation. Heat map and volcano plot analysis (Figures 2A,B) showed profound changes in the metabolic profile of myoblasts exposed to high CO_2_ compared to normal conditions. Pathway enrichment analyses revealed increased TCA cycle and carnitine pathway (Figure 2C), moreover many metabolites involved in these pathways were increased during high CO_2_ exposure, especially acyl carnitines (Figures 2D,E). To validate these results, we measured mitochondrial respiration using Seahorse technology, and found that basal, coupled and maximal mitochondrial respiration were higher in high CO_2_-exposed myoblasts (Figures 2F–I). All these data together are suggestive of increased TCA cycle due to increased β-oxidation.

### Increased Fatty Acid β-Oxidation by High CO_2_ Impairs C2C12 Myoblasts Differentiation Into Myotubes

**Figure 3:**
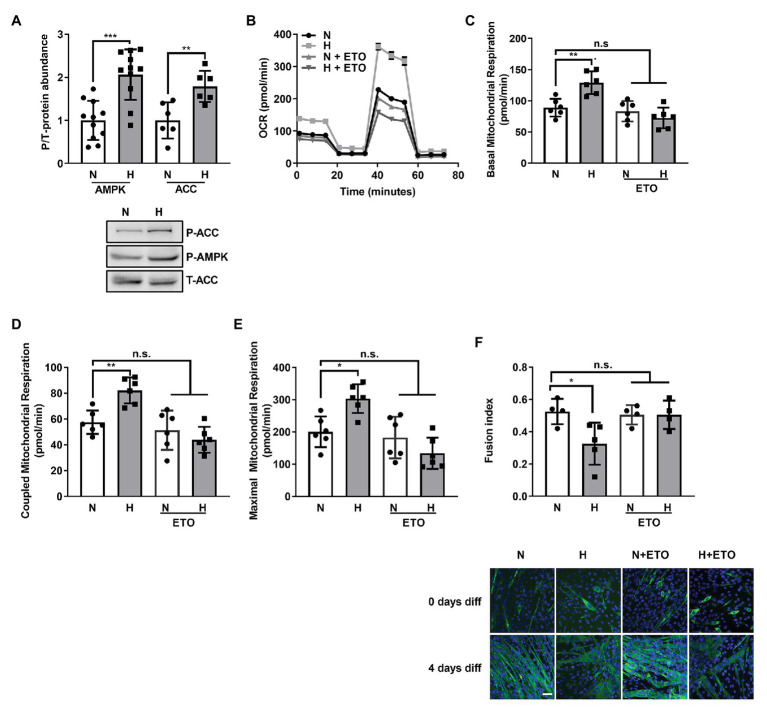
Increased fatty acid β-oxidation by high CO_2_ impairs C2C12 myoblasts differentiation into myotubes. **(A)** C2C12 myoblasts were exposed to normal (N) or high CO_2_ conditions (H) for 3 days, harvested, lysated, and protein expression analyzed by Western Blot. Upper panel: graph showing relative values of phosphorylated‐ vs. total-AMP-activated Kinase (AMPK) and Acetyl-CoA carboxylase (ACC; *n* = 6–10). Lower panel: representative Western blots. **(B–E)** Mitochondrial respiration was measured using Seahorse technology to detect the OCR values in C2C12 myoblasts exposed for 3 days to normal (N) or high CO_2_ (H) conditions in the absence or presence of 10 μM of the fatty acid import inhibitor etomoxir (ETO). **(B)** Graph shows a representative OCR after treatment with oligomycin, FCCP and rotenone. **(C)** Graph shows basal mitochondrial respiration. **(D)** Graph shows coupled mitochondrial respirationb. **(E)** Graph shows maximal mitochondrial respiration. **(F)** C2C12 myoblasts were exposed to normal (N) or high CO_2_ conditions (H) for 3 days in the absence or presence of 10 μM ETO and allowed to differentiate in normal CO_2_ conditions up to 4 days. Upper panel: graph showing fusion index (*n* = 4–5). Lower panel: representative immunofluoresecent images from C2C12 myoblasts at 4 days of differentiation showing MHC (green) expression and nuclei (blue). Scale bar: 100 μm. Data from **(A)** was analyzed by unpaired *t*-test. Data from **(C–F)** were analyzed by one-way ANOVA followed by Tukey’s multiple comparison test. ^*^p < 0.05; ^**^p < 0.01; ^***^p < 0.001; n.s: not significant.

A recent publication described a role for increased fatty acid β-oxidation in the atrophy of skeletal muscle in cancer (Fukawa et al., 2016). AMPK, a master regulator of metabolism (Herzig and Shaw, 2018), has been shown to be activated by high CO_2_ (Vadasz et al., 2008; Jaitovich et al., 2015) and plays a role in the regulation of fatty acid metabolism (O’Neill et al., 2013). As shown in Figure 3A, C2C12 myoblasts exposed to high CO_2_ have increased p-AMPK and increased ACC phosphorylation, a known AMPK substrate. ACC phosphorylation is known to indirectly increase carnitine palmitoyltransferase-1 (CPT1) activity and consequently β-oxidation (Wakil and Abu-Elheiga, 2009).

To further elucidate the role of fatty acid oxidation in the hypercapnia-impaired C2C12 myoblast differentiation, we exposed myoblasts to normal or high CO_2_ for 3 days in the presence or absence of the fatty acid import inhibitor etomoxir (Lopaschuk et al., 1988) and studied its effects on mitochondrial respiration using SeaHorse technology (Figure 3B). We found that 10 μM etomoxir blunted the hypercapnia effect in the increased basal, coupled and maximal mitochondrial respiration (Figures 3C–E). Moreover, etomoxir treatment also improved C2C12 myoblast differentiation into myotubes (Figure 3F). Taken together, these data suggest that fatty acid utilization during high CO_2_ exposure is a maladaptive metabolic response.

### Hypercapnia Impairs Muscle Regeneration, Which Is Restored by Etomoxir, in an *in vivo* Cardiotoxin Injury Model

**Figure 4:**
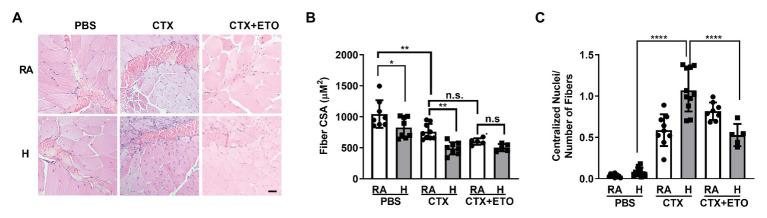
Hypercapnia impairs muscle regeneration, which is restored by etomoxir, in an *in vivo* cardiotoxin injury model. **(A)** Representative H&E images of tibialis anterior muscle from mice exposed to room air (RA) or high CO_2_ for 21 days (H) injected at day 0 with PBS, 10 μl of 10 μM cardiotoxin (CTX) with or without pre-treatement with 25 mg/kg ETO. Bar = 20 μm. **(B)** Graphs shows fiber cross sectional area (CSA) from mice exposed and treated as in (**A**; *n* = 5–8 mice per condition). **(C)** Graphs shows the amount of centralized nuclei per number of fibers from mice exposed and treated as in (**A**; *n* = 5–8 mice per condition). Data from **(B,C)** were analyzed by one-way ANOVA followed by Tukey’s multiple comparison test. ^*^p < 0.05; ^**^p < 0.01; ^****^p < 0.0001; n.s: not significant.

To interrogate whether hypercapnia impairs skeletal muscle repair *in vivo*, we used a well-established model of cardiotoxin injury (Ceco et al., 2014; Garry et al., 2016). We injured the left TA muscle by injecting cardiotoxin and used the right TA muscle injected with PBS as a control. We determined skeletal muscle remodeling after 21 days of exposing the mice to normoxic hypercapnia (10% CO_2_) and compared them to the mice in room air. Consistent with our previous study, we observed a decreased CSA in the TA muscle of mice exposed to high CO_2_ (Jaitovich et al., 2015; Figures 4A,B). Mice injected with cardiotoxin showed a further decrease in CSA when exposed to hypercapnia, which was prevented in mice treated with etomoxir (Figures 4A,B).

Once the skeletal muscle is injured, the stem cells are activated and begin proliferation and differentiation to repair the damage (Snijders et al., 2015; Laumonier and Menetrey, 2016). In the initial phase of repair, the nuclei localize in the center of the growing fiber, and muscle recovery is determined by nuclei localized at the muscle fiber periphery (Griffin et al., 2010; Forcina et al., 2020). In agreement with our *in vitro* data showing impaired myoblast differentiation, we found that mice exposed to hypercapnia had increased number of centralized nuclei (Figure 4C) which was prevented when mice were treated with etomoxir. Collectively, our data suggest that hypercapnia slows the repair of skeletal muscle by affecting fatty acid metabolism.

## Discussion

Loss of skeletal muscle mass is a feature of muscle dysfunction in patients with pulmonary diseases, such as COPD (Debigare et al., 2001; Wust and Degens, 2007; Gea et al., 2016; Barreiro and Jaitovich, 2018). The regulation of muscle mass is thought to result from the balance between protein synthesis and degradation. However, there is evidence that cell turnover is also involved in muscle growth and maintenance of muscle mass. Addition of new myonuclei due to the fusion of satellite cells, the stem cells of skeletal muscle, into pre-existing myotubes play a central role in muscle regeneration (Charge and Rudnicki, 2004; Yin et al., 2013). Hypercapnia is associated with worse outcomes in patients with COPD (Soler-Cataluna et al., 2005; Steer et al., 2010; Yang et al., 2015; Fazekas et al., 2018). We have previously reported that high CO_2_ induces muscle atrophy, through the activation of the E3 ubiquitin ligase *via* AMPKα2-FoxO3a-MuRF1 (Jaitovich et al., 2015). The main finding of the present study is that high CO_2_ also negatively affects muscle regeneration by impairing expansion, differentiation, and fusion of satellite cells into myotubes. We found that impaired differentiation was due to high CO_2_-induced AMPK phosphorylation and consequent increased fatty acid oxidation as the use of the CPT1 inhibitor etomoxir prevented it both *in vitro* and *in vivo*.

Satellite cells are responsible for regulating skeletal muscle repair by proliferating and differentiating into fusion-competent myoblasts and facilitating myofiber regeneration following injury (Lepper et al., 2011; Murphy et al., 2011; Tidball, 2011; Laumonier and Menetrey, 2016). Although our study has the limitation of not using primary satellite cells, we found that high CO_2_ decreased the proliferation rate of C2C12 myoblasts concording with a previous report in lung epithelial cells and fibroblasts (Vohwinkel et al., 2011). These results suggest a maladaptive effect of high CO_2_ in cell proliferation *via* cell-type specific mechanism, particulary targeting mitochondria, as in epithelial cells and fibroblasts, we found a decreased mitochondrial respiration (Vohwinkel et al., 2011), whereas in the present study, we found increased mitochondrial respiration in myoblasts. We also observed that C2C12 myoblasts exposed to high CO_2_ had decreased fusion index leading to impaired satellite cell differentiation, as highlighted by decreased MHC and NaK-ATPase α2 protein expression (Ladka and Ng, 2000; Tsukamoto et al., 2018). However, not all differentiation markers were affected as myogenin levels were not changed after high CO_2_ exposure, concordantly with previous publications reporting that not all markers of differentiation get modified in satellite cells with impaired differentiation (Fukawa et al., 2016). In agreement with previous publications, the impairment in proliferation and differentiation of C2C12 myoblasts was independent of cell death or pH (Briva et al., 2007; Vohwinkel et al., 2011; Bharat et al., 2020).

Changes in metabolism have been implicated in impaired satellite cells differentiation during cancer-mediated cachexia (Fukawa et al., 2016; Ceco et al., 2017). CO_2_ is a by-product of oxidative metabolism, and there are indications that high CO_2_ level has metabolic effects on epithelial and fibroblast cells (Vohwinkel et al., 2011). We performed metabolic profiling of C2C12 myoblasts exposed to normal CO_2_, compared them to myoblasts exposed to high CO_2_ for 3 days, and found that myoblasts exposed to high CO_2_ had changes in the TCA cycle, in agreement with our previous publication (Vohwinkel et al., 2011). Interestingly, we found an upregulation of carnitine and carnitine-derivatives. Moreover, we found this to be translated into increased mitochondrial respiration with an increase in oxygen consumption linked to ATP production and maximal respiration capacities. Increased fatty acid oxidation by satellite cells has been shown to be involved in the development of sarcopenia (Baraibar et al., 2016) and cancer-induced cachexia (Fukawa et al., 2016), both characterized by impaired satellite cell function. Our data suggest that high CO_2_ levels cause increased fatty acid oxidation in myoblasts, which could lead to impaired satellite cell function and muscle dysfunction, as seen in COPD patients. For instance, a significantly higher mitochondrial capacity is observed in the diaphragm muscle of patients with COPD as compared with control subjects (Levine et al., 2002).

AMPK is recognized as the cellular energy sensor and metabolic regulator. It has been linked to the pathogenesis of muscle atrophy, through augmented myofibrillar protein degradation by muscle atrophy F-box (MAFbx) and MuRF1 expression (Nakashima and Yakabe, 2007) and FoxO3a transcription factor-induced autophagy (Sanchez et al., 2012). We have reported that high CO_2_ activates AMPK in lung epithelial cells and myotubes (Vadasz et al., 2008; Jaitovich et al., 2015), and in the present report, we observed a high CO_2_-induced activation of AMPK in C2C12 myoblasts. ACC plays an essential role as a regulator of fatty acid metabolism by catalyzing acetyl-CoA carboxylation to form malonyl-CoA (Fullerton et al., 2013). ACC is a substrate of AMPK leading to the suppression of ACC activity (Carling et al., 1987), which leads to decreased levels of Malonyl CoA, an intermediate molecule, and an indirect negative modulator of carnitine palmitoyltransferase 1 (CPT1). CPT1 is a mitochondrial outer membrane enzyme that conjugates long chain fatty acyl CoAs to carnitine and facilitates uptake into the mitochondrial matrix for oxidation (Wakil and Abu-Elheiga, 2009). We not only found AMPK phosphorylation in C2C12 myoblasts exposed to high CO_2_ but also increased ACC phosphorylation, suggesting a mechanism by which high CO_2_ might increase fatty acid metabolism, leading to satellite cell dysfunction. AMPK seems to be a common denominator in high CO_2_-mediated muscle dysfunction. With regards to muscle regeneration, AMPKα1 plays a prominent role in regulating satellite cell dynamics, with AMPKα2 playing a potentially more important role in regulating muscle degradation during atrophy (Thomson, 2018). AMPK is activated in response to an increase in intracellular AMP and ADP levels that occurs in response to a fall in ATP. However, AMPK is also activated by an increase in intracellular calcium ions, which is mediated by Ca^2+^/calmodulin-dependent kinase kinase β (CaMKK-β; Steinberg and Carling, 2019). We have previously shown that high CO_2_-induced AMPK activation was due to increased intracellular Ca^2+^ in alveolar epithelial cells (Vadasz et al., 2008), making it a possible mechanism in C2C12 myoblasts, as we found increased mitochondrial respiration.

We demonstrated that high CO_2_ increased fatty acid oxidation in satellite cell and dysfunction by pre-treating the C2C12 myoblasts with the CPT1 inhibitor etomoxir (Lopaschuk et al., 1988). Etomoxir prevented both high CO_2_-increased mitochondrial respiration as well as decreased myoblast fusion in agreement with the work of Fukawa et al. (2016) in cancer-induced cachexia. Moreover, we found etomoxir to be an effective inhibitor *in vivo* when using the cardiotoxin-mediated skeletal muscle injury model. Recently, several reports have challenged the specificity of etomoxir as a CPT1 inhibitor (O’Connor et al., 2018; Raud et al., 2018); therefore, further investigation in the role of fatty acid metabolism in C2C12 exposed to high CO_2_ levels is warranted.

We found that the mice exposed for up to 21 days to high CO_2_ not only had decreased CSA (Jaitovich et al., 2015) but also increased the number of centralized nuclei. Centralized nuclei are markers of muscle diseases and delayed repair (Griffin et al., 2010; Forcina et al., 2020). Cardiotoxin causes muscle damage affecting both CSA and centralized nuclei (Ceco et al., 2014), and we found that mice exposed to high CO_2_ recovered slower after cardiotoxin damage, reflected by decreased myofiber CSA and increased the number of centralized nuclei. Interestingly, intraperitoneal etomoxir was enough to restore the hypercapnia-induced effects on muscle regeneration, given by normalization of centralized nuclei; however, there was an absence of recovery of the CSA, which might be explained by the persistent atrophy induced by proteolysis mediated activation of E3 Ligases of mature myotubes (Jaitovich et al., 2015). These data are suggestive of the pleiotropic effects of high CO_2_ on muscle pathophysiology: it causes skeletal muscle atrophy through the activation of the E3 ubiquitin ligase MuRF1 *via* AMPKα2-FoxO3a (Jaitovich et al., 2015) and delays regeneration *via* the inhibition of proliferation and differentiation of satellite cells.

In summary, we provide evidence that high CO_2_ impairs skeletal muscle myoblasts differentiation by increased fatty acids oxidation and mitochondrial respiration overload. Accordingly, we propose a mechanism by which a subset population of COPD patients with hypercapnia might have impaired skeletal muscle recovery after injury. A better understanding of these mechanisms might inform novel therapies to prevent and improve skeletal muscle atrophy in COPD patients to decrease morbidity and improve quality of life.

## Data Availability Statement

The original contributions presented in the study are included in the article/Supplementary Material, further inquiries can be directed to the corresponding authors.

## Ethics Statement

The animal study was reviewed and approved by Northwestern University Institutional Animal Care and Use Committee (#IS00000245).

## Author Contributions

EC and DC contributed to conceptualization, study design, methodology, data collection, formal analysis, and manuscript writing. SW, MS, and LW contributed to formal analysis, methodology, and drafting the manuscript. LV conducted experiments. AB contributed to conceptualization and writing. NC contributed to conceptualization, study design, data curation, writing, and funding acquisition. EL contributed to conceptualization, study design, methodology, data collection, validation, formal analysis, investigation, data curation, writing, visualization, and supervision. JS contributed to conceptualization, study design, resources, data curation, writing, supervision, project administration, and funding acquisition. All authors contributed to the article and approved the submitted version.

### Conflict of Interest

The authors declare that the research was conducted in the absence of any commercial or financial relationships that could be construed as a potential conflict of interest.
